# *QuickStats*: Percentage* of Adults Aged ≥18 Years in Fair or Poor Health,^†^ by Family Income^§^ and Age Group — National Health Interview Survey, United States, 2021^¶^

**DOI:** 10.15585/mmwr.mm7213a6

**Published:** 2023-03-31

**Figure:**
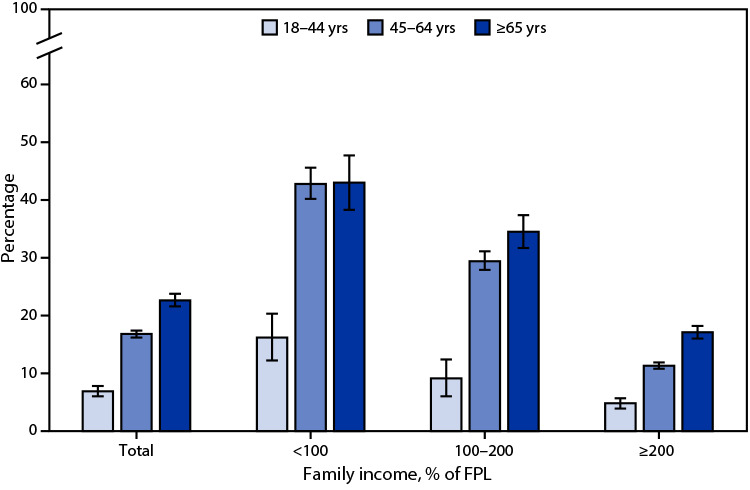
In 2021, 13.6% of adults aged ≥18 years assessed their health as fair or poor. The percentage increased with age from 6.9% for those aged 18–44 years, to 16.8% for those aged 45–64 years, and 22.6% for those aged ≥65 years. The same pattern of increasing percentages with age was found for adults living in families with incomes 100% to <200% of FPL and ≥200% of FPL. For adults living in families with incomes <100% of FPL, the percentage in fair or poor health was lowest among those aged 18–44 years (16.2%), but similar among adults aged 45–64 years (42.8%) and those aged ≥65 years (43.0%). The percentage of adults in fair or poor health decreased with increasing incomes for each age group.

**Source:** National Center for Health Statistics, National Health Interview Survey, 2021. https://www.cdc.gov/nchs/nhis/index.htm

